# Altered GABA Concentration in Brain Motor Area Is Associated with the Severity of Motor Disabilities in Individuals with Autism Spectrum Disorder

**DOI:** 10.1007/s10803-020-04382-x

**Published:** 2020-01-30

**Authors:** Yumi Umesawa, Kanae Matsushima, Takeshi Atsumi, Toshihiro Kato, Reiko Fukatsu, Makoto Wada, Masakazu Ide

**Affiliations:** 1 Department of Rehabilitation for Brain Functions, Research Institute of National Rehabilitation Center for Persons with Disabilities, 4-1, Namiki, Tokorozawa-shi, Saitama, 359-8555 Japan; 2 Department of Human Health Sciences, Graduate School of Medicine, Kyoto University, Kyoto-shi, Kyoto, Japan; 3 Japan Society for the Promotion of Science, Chiyoda-ku, Tokyo, Japan; 4 Present Address: Kansai Medical University, Hirakata-shi, Osaka, Japan; 5 Present Address: Department of Medical Physiology, Faculty of Medicine, Kyorin University, Mitaka-shi, Tokyo, Japan

**Keywords:** Developmental coordination disorder, Autism spectrum disorder, Gamma-aminobutyric acid, Magnetic resonance spectroscopy, Primary motor area, Supplementary motor area

## Abstract

Several motor disabilities accompanied with autism spectrum disorder (ASD) are widely known despite limited reports of underlying neural mechanisms. Gamma-aminobutyric acid (GABA) levels in the motor-related cortical areas modulate several motor performances in healthy participants. We hypothesized that abnormal GABA concentrations in the primary motor area (M1) and supplementary motor area (SMA) associate with different motor difficulties for ASD adolescents/adults. We found that increased GABA concentrations in M1 measured using ^1^H-magnetic resonance spectroscopy exhibited lower motor performance in tasks requiring increased muscle strength while lower GABA concentrations in SMA were associated with lower scores in tests measuring body coordination. The degrees of neural inhibition in the M1 and SMA regions would contribute to different dimensions of motor disabilities in autism.

**Electronic supplementary material:**

The online version of this article (10.1007/s10803-020-04382-x) contains supplementary material, which is available to authorized users.

Several kinds of motor disabilities are frequently accompanied by other developmental disorders, such as autism spectrum disorder (ASD), learning disorder, and attention deficit hyperactivity disorder. Individuals with ASD have been found to have slower and less accurate manual dexterity (Green et al. 2002; Manjiviona and Prior 1995), poor ball skills (Miyahara et al. 1997; Staples and Reid 2010), and poor balance (Freitag et al. 2007; Jansiewicz et al. 2006). A demographic study in the United Kingdom reported that Developmental Coordination Disorder (DCD) was present in 79% of children with ASD (Green et al. 2009). DCD has been defined by the Diagnostic and Statistical Manual of Mental Disorders, fifth edition (DSM-5), issued by the American Psychiatric Association (APA 2013). DCD is diagnosed if patients exhibit motor skill learning and performance that is below expected levels for their age. DCD is characterized by clumsiness (e.g., dropping things, bumping into others), lateness, and inaccuracy of motor skills (e.g., catching, cutting with scissors or with a knife, writing, riding a bicycle, sports). Barnhart et al. (2003) suggested that DCD becomes apparent during school-age (6–12 year-old); before that, DCD remains difficult to diagnose through the DSM-5 (APA 2013). In addition, a 10-year follow-up study of children who exhibited clumsiness found that motor problems persisted at later developmental stages (Losse et al. 1991).

These motor disabilities in individuals with ASD represented by DCD includes several aspects that appear in daily life. To address the underlying neural substrates of the disrupted motor function in ASD [such as the impairments of the internal model of motor control (Adams et al. 2014)] researchers have focused on specific behaviors in restricted experimental situations (e.g., reaching and grasping). This internal model works as a predictive system that enables quick modifications of one’s own motion by comparing actual sensory feedback with the predicted sensory feedback from the efference copy (Blakemore et al. 1998; Kawato 1999). The cerebellum has been implicated as an important region in the acquisition of an internal model (Diedrichsen et al. 2007; Smith and Shadmehr 2005); furthermore, several studies have reported that individuals with ASD exhibit cerebellum abnormalities, that is, reduced Purkinje cell size (Fatemi et al. 2002; Ritvo et al. 1986), a qualitative decrease in cerebellar granule cell density (Kemper and Bauman 1993), and hypoplasia of cerebellar vermal lobules (Courchesne et al. 1988). The prism adaptation task has been frequently used to evaluate the impairment of the internal model and requires the participants to adjust their reaching motion of the hand when they wear prism glasses. One study has suggested that children with ASD could adapt their motion similar to those of neurotypical children (Gidley Larson et al. 2008). Another potential neural basis of DCD is a dysfunction of the mirror neuron system (Reynolds et al. 2015). This system involves a neural network between the inferior part of the parietal and ventral part of the premotor cortex (Rizzolatti et al. 2001) and underlies the association between imitation and understanding the intentions of others’ behavior. One electroencephalography study found that neural activity in the motor area did not increase during observation of another’s motion in individuals with ASD, in contrast to the greater activation seen in typically developing (TD) individuals (Oberman et al. 2005). Despite many reports of abnormal neural circuits in individuals with ASD, their association with DCD has not yet been investigated.

Molecular-level cortical abnormalities have also been reported in ASD. One suggested characteristic feature of the ASD brain is the severe excitability/inhibitory imbalance caused by alterations in gamma-aminobutyric acid (GABA) levels (Pizzarelli and Cherubini 2011). GABA is a major cortical inhibitory neurotransmitter that depresses neural activity in the cerebral cortex (Krnjević and Schwartz 1967). Recent years have seen technical developments of ^1^H-magnetic resonance spectroscopy (^1^H-MRS), which is a non-invasive neuroimaging method that can estimate concentrations of specific chemical metabolites and neurotransmitters in humans in vivo (Jansen et al. 2006). Using ^1^H-MRS, a reduced ratio of GABA+ to creatine (Cr) or H_2_O in the primary motor area (M1) (partially including the somatosensory area [S1]) was demonstrated in children with ASD relative to TD controls (Gaetz et al. 2014; Puts et al. 2017). The measured GABA concentrations in these publications, as well as in the current study, included macromolecules, thus, it is expressed as “GABA+” throughout the manuscript. It was also reported that the ratio *N*-acetyl aspartate (NAA)/Cr was decreased whereas the Choline (Cho)/Cr ratio was increased in the frontal lobe of children with ASD (Margari et al. 2018). One systematic review of studies using ^1^H-MRS metabolite measurements in the brains of individuals with ASD indicated reduced levels of GABA+, NAA, glutamate/glutamine (Glx), Cr, and Cho in children with ASD as opposed to inconsistent results in adults with ASD (Ford and Crewther 2016). Several studies with TD individuals have also shown the important contribution of GABA+ levels in M1 in relation to motor performance. For example, participants with a lower ratio of GABA+/NAA in M1 tended to show shorter reaction times in a visually cued sequence task performed with four fingers (Stagg et al. 2011). GABA+ concentrations in the sensorimotor cortex measured by ^1^H-MRS were quickly decreased after a motor learning task which required participants to adjust the hand force to the target force, which was indicated visually on the screen (Floyer-Lea et al. 2006). Anodal transcranial direct current stimulation (a-tDCS) was reported to affect neurotransmitter levels and reduce GABA+ levels in the cerebral cortex compared with those before the stimulation (Kim et al. 2014; Stagg et al. 2009). It was suggested that reduced activity of glutamic acid decarboxylase (GAD)67, the rate-limiting enzyme in the major metabolic pathway for GABA synthesis in the human cortex, was involved in this reduction of GABA (Stagg et al. 2009). Application of a-tDCS over M1 has been found to enhance the maximal pinch force on the leg (Tanaka et al. 2009), and a-tDCS stimulation also increased the isometric force endurance of elbow flexor muscles with an increased corticospinal excitability (Cogiamanian et al. 2007). Furthermore, delivering a-tDCS over M1 four times per week for two weeks improved the jumping force and coordination performance in ski athletes (Reardon 2016).

Previous findings suggest that individuals with a lower GABA+ concentration ratio in M1 tend to exhibit a better motor performance. A lower GABA+ concentration in M1 is linked to increased neuronal activity (Stagg et al. 2011); this might explain the observed improvement in motor performance. However, the association between motor skills required for daily life activities and GABA+ levels remains unclear. Indeed, studies reported that M1 activity influences motor performances testing somewhat unusual motor actions restricted to experimental situations, such as the pinch force of the thumb and index fingers on the leg (Tanaka et al. 2009) and isometric force endurance of elbow flexor muscles (Cogiamanian et al. 2007). Moreover, it seems paradoxical that a lower level of motor performance was observed in individuals with ASD while it was also reported that they had a reduced GABA+ concentration in M1. Those reports evaluated the motor performance using clinical assessments that cover several aspects of motor dysfunction relating to motor skills required in daily life activities of individuals with ASD (Green et al. 2009). The different approaches used to evaluate motor skills could explain the contradictory results found about the relationship between GABA+ concentration and motor impairment in individuals with ASD.

To reveal the neural basis of motor impairments in individuals with ASD, especially for characteristics studied using clinical assessments that evaluate several aspects of DCD, we examined types of motor skills associated with lower/higher GABA+ concentrations in brain motor areas. For this objective, we applied a clinical assessment tool which is used to evaluate both of fine and gross motor skills, the Bruininks-Oseretsky Test of Motor Proficiency, second edition (BOT-2, Pearson, San Antonio, USA; for more details, see the method section). The BOT-2 is widely adopted in clinical situations for DCD and evaluates motor skills that reflect motor performance in daily life (e.g., picking up coins, handling a ball, maintaining posture, and lifting the body by the arms). We used ^1^H-MRS to measure GABA+ concentrations of the left M1 and supplementary motor area (SMA), which contributes to movement coordination (Cattaneo et al. 2007; Mita et al. 2009). We aimed to elucidate the different contributions of GABA in representative brain motor areas (i.e., M1 and SMA) to different aspects of motor performances in ASD. Our results provide important clues for solving the question of the neural basis of motor impairments in ASD raised by a lot of studies using different research procedures as discussed above.

## Methods

### Participants

**Fig. 1:**
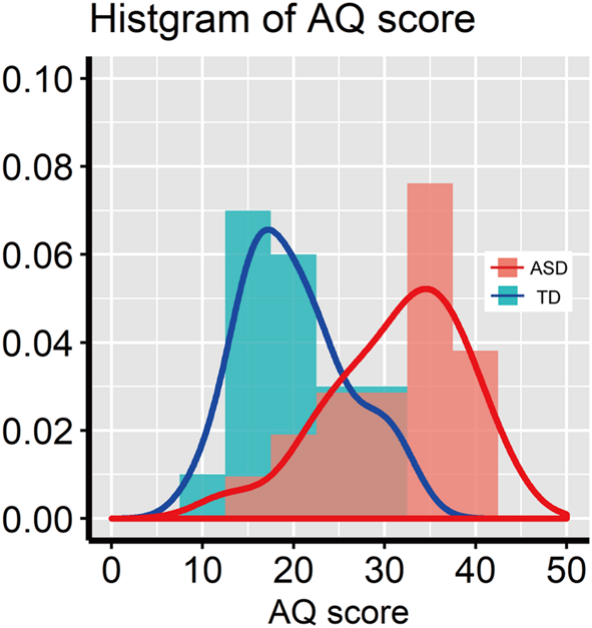
Distribution of AQ scores (Baron-Cohen et al. 2001; Wakabayashi et al. 2004) in the ASD and TD groups. Red and blue bars indicate ASD and TD participants, respectively. Solid lines denote the probability density function

We recruited 21 individuals with ASD (mean age: 19.2 ± 2.9 years, range 15–25 years; 6 females) and 23 TD individuals. Data from 3 TD participants were excluded from the final analysis because they had received special sports training, resulting in a final enrolment of 20 TD participants (mean age 19.0 ± 1.3 years, range 16–21 years; 6 females; Table 1). There were no group differences in the age and sex distributions. Individuals with a clinical diagnosis of ASD were recruited from parent groups for individuals with developmental disorders and the Department of Child Psychiatry in the hospital of the National Rehabilitation Center for Persons with Disabilities. Participants also completed the Japanese version of the Autism Spectrum Quotient (AQ) scale (Baron-Cohen et al. 2001; Wakabayashi et al. 2004), in which higher scores indicate stronger autistic traits. AQ scores in the ASD group were significantly higher than those in the TD group (two-tailed *t*-test: *t* (39) = 5.42, *p* < 0.001, Cohen’s *d* = 3.00, 95% confidence interval (CI) [7.10, 15.56]). The distribution of the AQ scores for all participants is illustrated in Fig. 1. The Intelligence Quotients (IQs) were assessed using the Wechsler Adult Intelligence Scale-Third Edition (WAIS-III). The Wechsler Intelligence Scale for Children-Fourth Edition (WISC-IV) was used to evaluate IQs for one 15-year-old participant (Subj1) and one 17-year-old participant (Subj2) who had taken the IQ test three years before the experiment. Since WISC-IV does not provide verbal IQ (VIQ) and performance IQ (PIQ), which are provided using WAIS-III, we did not include the two participants (Subj1 and 2) in the group comparisons analysis of VIQ and PIQ, except in the full-scale intelligence quotient (FIQ). The Wechsler Intelligence Scale for Children-Third Edition (WISC-III) was used to evaluate one 15-year-old participant (Subj3) (for more details, see Supplementary Tables 1 and 2). All ASD and TD participants presented FIQ values above that used to diagnosis an intellectual disorder (FIQ = 75), although statistically significant differences between the ASD and TD groups were found (*t* (39) = − 2.58, *p* = 0.014, *d* = 0.81, 95% CI  [− 18.61, − 2.24]). There were no significant between-group differences in age, VIQ and PIQ without two ASD participants whose IQs were evaluated using WISC- IV. Handedness was assessed using the laterality quotient score of the Edinburgh Handedness Inventory (Oldfield 1971), and we confirmed no group difference. When asked, we discovered that out of all participants, eight ASD participants were taking medical treatments (medical treatments of ASD participants are listed in Supplementary Table 3 and Fig. 1). All participants and their parents gave written informed consent after the study procedures had been fully explained. The present study was approved by the Ethics Committee of the National Rehabilitation Center for Persons with Disabilities.

**Table 1:** Information of the participants

	ASD group	TD group
Sex (M:F)	15:6 (N = 21)	14:6 (N = 20)
Age in years, mean (range)	19.2 (15–25)	19.0 (16–21)
LQ, mean (range)	72.4 (− 53 to 100)	76.3 (− 60 to 100)
AQ, mean (range)**	31.4 (13–42)	20.1 (10–32)
VIQ, mean (range)	112.2 (95–140)	115.3 (98–136)
PIQ, mean (range)	104.4 (72–140)	113.3 (80–137)
FIQ, mean(range)*	105.5 (78–135)	115.9 (93–131)

The AQ scores were evaluated using the Autism Spectrum Quotient (AQ) scale. The LQ scores were assessed using the Edinburgh Handedness Inventory (Oldfield 1971). The intellectual quotients (IQs) were assessed using the Wechsler Adult Intelligence Scale-Third Edition (WAIS-III)

*M* male, *F* female, *ASD* autism spectrum disorder, *TD* typically developing, *LQ* laterality quotient, *AQ* autism spectrum quotient, *VIQ* verbal intelligence quotient, *PIQ* performance intelligence quotient, *FIQ* full-scale intelligence quotient

*p < 0.05, **p < 0.01

### Procedures

A total of 12 out of 21 ASD participants and 15 out of the 20 TD participants completed the motor skills assessment and ^1^H-MRS data acquisition on the same day, whereas the remaining participants (9 ASD and 5 TD participants) completed these on different days. The average time values between the tasks were 68 ± 119 days (range 1–367 days) and 138 ± 287 days (range 2–721 days) in the ASD and TD groups, respectively. These values were not significantly different between groups (*t* (13) = − 0.67, *p* = 0.517, *d* = 0.32, 95% CI [− 299, 158]).

#### Assessment of Motor Skills

BOT-2 is the most widely used standardized measure of motor skills that possibly contribute to DCD. BOT-2 originates from the Bruininks-Oseretsky Test of Motor Proficiency which is composed of two components (gross motor and fine motor skills) (Crowe 1989; Robert and Brett 2005); the BOT-2 consisting of four subcategories was developed to measure motor difficulties in more detail. The assessed fine motor skills include precise bodily control that requires finger and hand movement based on visuomotor integration (*Fine manual control*) and bimanual/arm-hand coordination (*Manual coordination*). The gross motor skills assessed by the BOT-2 include maintaining posture, sequential and simultaneous bodily coordination (*Body coordination*), and strength of the trunk, as well as of the upper and lower body (*Strength and agility*). We measured each score in four subcategories (*Fine manual control, Manual coordination, Body coordination, and Strength and agility*). Each of the four subcategories consists of two subtests (eight subtests and 53 items in total) as follows: fine motor precision (7 items) and fine motor integration (8 items) are included in ‘*Fine manual control*’, manual dexterity (5 items) and upper limb coordination (7 items) are included in ‘*Manual coordination*’, bilateral coordination (7 items) and balance (9 items) are included in ‘*Body coordination*’, and running speed and agility (5 items) and strength (5 items) are included in ‘*Strength and agility*’. It took about 60 min to complete the assessment.

Since the BOT-2 has not been standardized in Japan yet, we calculated standardized scores from raw scores of all participants using the following formula:

$$T_{i} = \frac{{10\left( {x_{i} - u_{x} } \right)}}{{\sigma_{x} }} + 50$$

where *x*_*i*_ represents the sampled score. The *u*_*x*_ and *σ*_*x*_ correspond to the arithmetic mean and standard deviation calculated from each raw score of the four subcategories (*Fine manual control*, *Manual coordination*, *Body coordination,* and *Strength and agility*). The total score was also obtained by summarizing the standardized scores in the four subcategories.

#### MR Data Acquisition

**Fig. 2:**
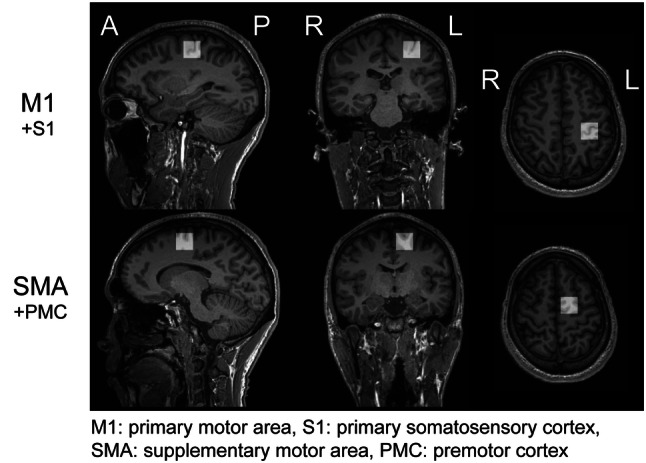
ROIs for ^1^H-MRS data acquisition. ROIs were placed in the left M1 and left SMA with a voxel size of 20 × 20 × 20 mm^3^. The M1 ROI was placed in the left precentral sulcus, and the midpoint of the SMA ROI was set as the upper part of the Brodmann area 6. The ROIs in the M1 and SMA areas included slightly the primary somatosensory cortex (S1) and premotor cortex (PMC), respectively. For both ROIs, the inclusion of bone and cerebrospinal fluid was avoided

MR scans were acquired using a 3 T MRI (MAGNETOM Skyra; Siemens AG, Munich, Germany) equipped with a 64-channel head coil. Before the ^1^H-MRS scanning, T1 images were obtained by a 3D Magnetization-Prepared Rapid Gradient-Echo sequence (number of slices = 224, slice thickness = 1 mm, repetition time [TR] = 2300 ms, echo time [TE] = 2.98 ms, flip angle = 9°) for the exact positioning of the regions of interest (ROIs) in a voxel size of 20 × 20 × 20 mm^3^. ROIs were the left M1 and left SMA (Fig. 2). The M1 ROI included the “hand-knob” of the left central sulcus (Yousry et al. 1997), and the SMA ROI was the upper part of the Brodmann area 6. The M1 and SMA ROIs slightly overlapped with the primary somatosensory cortex (S1) and premotor cortex (PMC), respectively. Thus, we represent in the figures the ROIs in the M1 and SMA regions as “M1 + S1” and “SMA + PMC”, respectively (see Fig. 2). We avoided the inclusion of bone and cerebrospinal fluid in the ROIs. Brain metabolites were obtained using the MEGA-PRESS spectral editing sequence (TR = 2000 ms, TE = 70 ms, 128 pairs of interleaved spectra, acquisition time < 9 min per ROI). Even though TE = 68 ms has been broadly accepted (Mullins et al. 2014; Rothman et al. 1993), we used a TE = 70 ms since the GABA spectra could be obtained with good quality in our experimental situation. Furthermore, TE = 70 ms has been reported to successfully acquire GABA signal using MEGA-PRESS in Siemens system (Hattingen et al. 2014; Sanaei Nezhad et al. 2018; Vega et al. 2014). A frequency-selective editing pulse was applied at 1.9 ppm (ON) and 7.5 ppm (OFF) in alternating spectral lines to differently refocus the GABA triplet signal at 3.02 ppm. In total, the measurements took 60 min including instruction, T1 acquisition, and ^1^H-MRS scanning. We carefully instructed participants not to move their head and keep their eyes closed during the sequence.

#### ^1^H-MRS Analysis

GABA+ concentrations were quantified from Siemens RDA file (spectra averaged on the scanner) using the Gannet 2.0 software (Edden et al. 2014) with MATLAB (R2016b, Mathworks, USA) on a workstation PC (Dell Precision T3620, Dell, Japan). In the Gannet 2.0 analysis, raw time-domain data from the scanner were frequency- and phase-corrected using spectral correction (Near et al. 2015) and filtered with 3-Hz exponential line broadening to maximize the quality of the edited spectrum. Fast Fourier transform was applied to the data to convert it from time- to frequency-domain data with zero-filling up to 32 k points. The GABA+ signal was calculated by subtracting two spectra (one applied frequency-selective editing pulses, the other not) to separate the GABA+ signal from the strong overlying Cr signal. The edited GABA+ signal was fitted using a five-parameter Gaussian model (Gaussian amplitude, Gaussian width, baseline offset, baseline gradient, and frequency) from 2.79 to 3.55 ppm. The Cr and water signals were fitted using a six-parameter Lorentzian model and a Gaussian–Lorentzian model, respectively. The GABA+ fit error was defined as the standard deviation (SD) of the residuals expressed as a percentage of the signal height. The GABA+ concentration is expressed in institutional units (iu) relative to water and as an integral ratio relative to Cr in a primary outcome measure. GABA+ concentrations were corrected for tissue fractions using the segmentation and quantification steps implemented in Gannet 2.0 (Harris et al. 2015). These steps included tissue segmentation in SPM (SPM12, Wellcome Trust Center for Neuroimaging) to estimate the fractions in gray matter, white matter, and cerebrospinal fluid.

### Statistical Methods

The Student’s *t-*test was used to compare group means of BOT-2 scores and tissue-corrected GABA+ concentrations in M1 and SMA. We calculated Cohen’s *d* to indicate effect sizes in group differences. We performed partial correlation analyses to test the association of the variables while controlling FIQ score effects because of a significant group difference in the FIQ. Bonferroni correction for multiple comparisons of scores in the four subcategories in BOT-2 assessment (*Fine manual control*, *Manual coordination*, *Body coordination*, *Strength and agility*) was applied to be adjusted alpha threshold of *p* < 0.0125. Our sample size of the correlation analysis was determined based on previous studies that investigated the association between GABA+ concentrations and behavioral tasks with a *p*-value below 0.05 and a post hoc power above 0.7 (Bhattacharyya et al. 2013; Blicher et al. 2014; Boy et al. 2010; Heba et al. 2015; Kim et al. 2014; Stagg et al. 2011). However, our sample sizes (N = 21 and 20 in ASD and TD groups, respectively) were not enough even if we took these studies into consideration, we performed permutation based on Pearson’s correlation coefficients between BOT-2 scores and GABA+ concentrations. For all analysis, 10,000 permutations were used to estimate the distribution of the null hypothesis and implemented as described (Groppe et al. 2011). We calculated the confidence intervals of partial correlation coefficients based on Fisher’s *z* transformation. We used SPSS Version 23.0 (IBM, New York, U.S.) to perform the *t*-tests and analyze the partial correlations, G*power 3.1 (Erdfelder et al. 1996) to calculate the effect sizes, and R (R Core Team 2018) to perform permutation test.

## Results

### BOT-2 Results

**Fig. 3:**
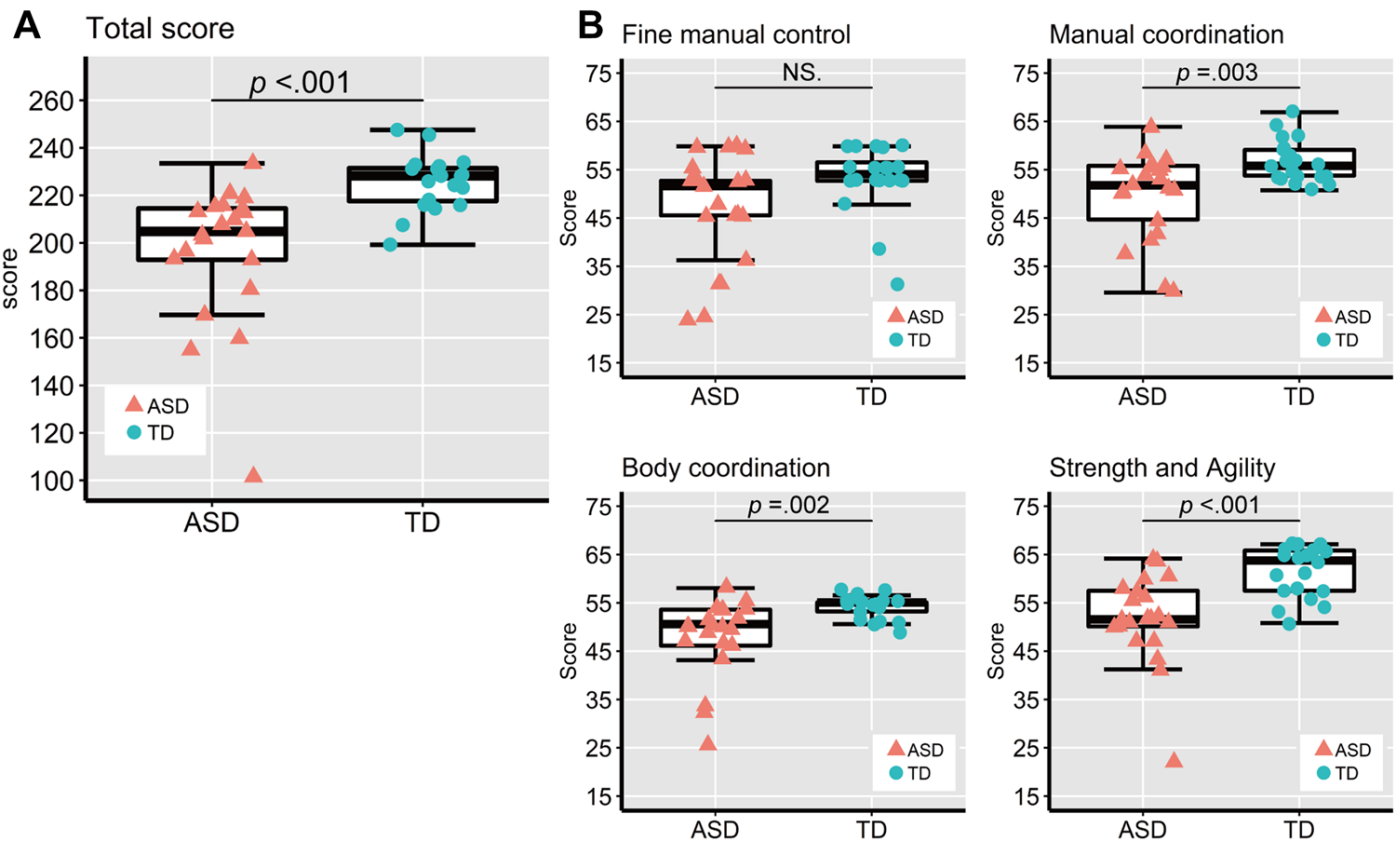
BOT-2 scores. **a** BOT-2 total score in the ASD and TD groups. The upper and lower boundaries of the standard boxplots represent the 25th and 75th percentiles. The horizontal line across the box marks the median of the distribution. The vertical lines below and above the box represent the minimum and maximum values, respectively. **b** BOT-2 scores in the four subcategories in the ASD and TD groups. Bonferroni correction for multiple comparisons was applied to the Student’s t-test analysis (adjusted alpha *p* < 0.0125)

BOT-2 total scores and subcategory scores for the ASD and TD groups are shown in Fig. 3. The total score in the ASD group was significantly lower than that of the TD group (*t* (39) = − 4.15, *p* < 0.001, Cohen’s *d* = 1.31, 95% CI [− 43.81, − 15.1]). The ASD group also had significantly lower scores in all subcategories compared to the TD group (*Manual coordination*: *t* (39) = − 3.17, *p* = 0.003, *d* = 1.00, 95% CI [− 11.57, − 2.55]; *Body coordination*: *t* (39) = − 3.32, *p* = 0.002, *d* = 1.05, 95% CI [− 10.12, − 2.46]; *Strength and agility*: *t* (39) = − 4.26, *p* < 0.001, *d* = 1.34, 95% CI [− 14.69, − 5.22]) except in the *Fine manual control* subcategory (*t* (39) = − 2.01, *p* = 0.045, *d* = 0.65, 95% CI [− 12.14, − 0.14]). Supplementary Fig. 2 shows the boxplots of the eight subtest scores.

### ^1^H-MRS Results

**Fig. 4:**
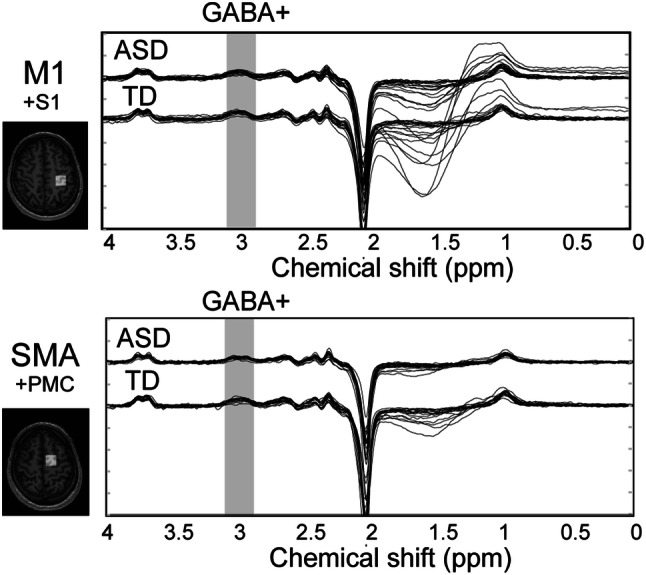
MEGA-PRESS spectra in the left M1 and left SMA. The horizontal axis shows the chemical shift of the resonance frequency. The MEGA-PRESS spectra for M1 (upper graph) and SMA (lower graph) are shown. Each solid line indicates each participant’s spectrum, for both ASD and TD groups. The grey-shaded range indicates 3 ppm where the GABA+ concentration is reflected

**Fig. 5:**
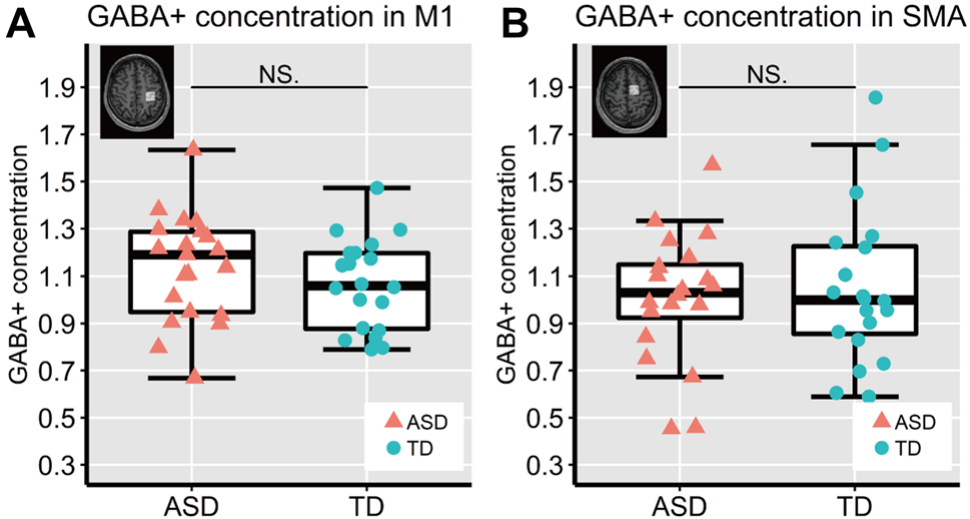
Tissue-corrected GABA+ concentrations in M1 (**a**) and SMA (**b**) in the ASD and TD groups

The MEGA-PRESS spectra in the M1 and SMA overlapped between the ASD and TD groups (Fig. 4). Tissue fractions did not differ between groups in the M1 ROI and the SMA ROI (Table 2). We confirmed that each of the fit errors for the GABA+ measurements in both ROIs was below 20%, a criterion used in previous reports as the acceptance level to obtain reliable MRS measurements (Brix et al. 2015; Harada et al. 2011), although the averaged fitting error in the ASD group was significantly greater than that in the TD group only in SMA ROI (*t* (38) = 3.1, *p* = 0.003, *d* = 1.00, 95% CI [0.008, 0.037]; Table 3). There were no group differences about full-width half-maximum of the Cr or GABA+ linewidth (Cr FWHM/ GABA+ FWHM) and the SD of the water frequency in Hz in any ROIs. Tissue-corrected GABA+ concentrations in the SMA of one ASD participant were excluded from subsequent analyses because these values exceeded three times the SD of the mean (Nakai and Okanoya 2016). There were no significant between-group differences in the GABA+ concentrations in the M1 or SMA (M1: *t* (39) = 1.09, *p* = 0.281, *d* = 0.34, 95% CI [− 0.06, 0.20]; SMA: *t* (38) = − 0.44, *p* = 0.665, *d* = 0.14, 95% CI [− 0.24, 0.15]; Fig. 5). For the same participants, we also quantified GABA+ concentrations relative to Cr and H_2_O without tissue corrections and obtained similar results (see Supplementary Tables 4 and 5).

**Table 2:** Tissue fractions in M1 and SMA ROIs

	ASD group	TD group
M1 ROI		
Gray matter (%)	42 (± 6)	42 (± 4)
White matter (%)	48 (± 8)	47 (± 5)
Cerebrospinal fluid (%)	10 (± 2)	10 (± 3)
SMA ROI		
Gray matter (%)	44 (± 5)	47 (± 4)
White matter (%)	50 (± 7)	47 (± 5)
Cerebrospinal fluid (%)	6 (± 2)	7 (± 2)

*ASD* autism spectrum disorder, *TD* typically developing, *M1* primary motor area, *SMA* supplementary motor area, *ROI* region of interest

**Table 3:** Metrics of data quality for MRS measurements in M1 and SMA ROIs

	ASD group	TD group
M1 ROI		
Cr FWHM (Hz)	10.1.(± 2.1)	9.6 (± 1.6)
GABA + FWHM (Hz)	21.5.(± 2.7)	20.0 (± 2.0)
Water frequency SD (Hz)	0.03 (± 0.04)	0.03 (± 0.03)
GABA fit error (%)	8.4 (± 2.4)	7.6 (± 1.6)
SMA ROI		
Cr FWHM (Hz)	9.5.(± 1.7)	10.4 (± 1.7)
GABA + FWHM (Hz)	21.8.(± 5.3)	21.7 (± 3.4)
Water frequency SD (Hz)	0.02 (± 0.03)	0.01 (± 0.02)
GABA fit error (%)**	10.0 (± 2.8)	7.8 (± 1.5)

*ASD* autism spectrum disorder, *TD* typically developing, *M1* primary motor area, *SMA* supplementary motor area, *ROI* region of interest, *Cr FWHM* the full-width half-maximum of the creatine peak, *Cr FWHM* the full-width half-maximum of the gamma-aminobutyric acid peak

**p < 0.01

### Correlation Between BOT-2 Scores and GABA+ Concentrations

**Fig. 6:**
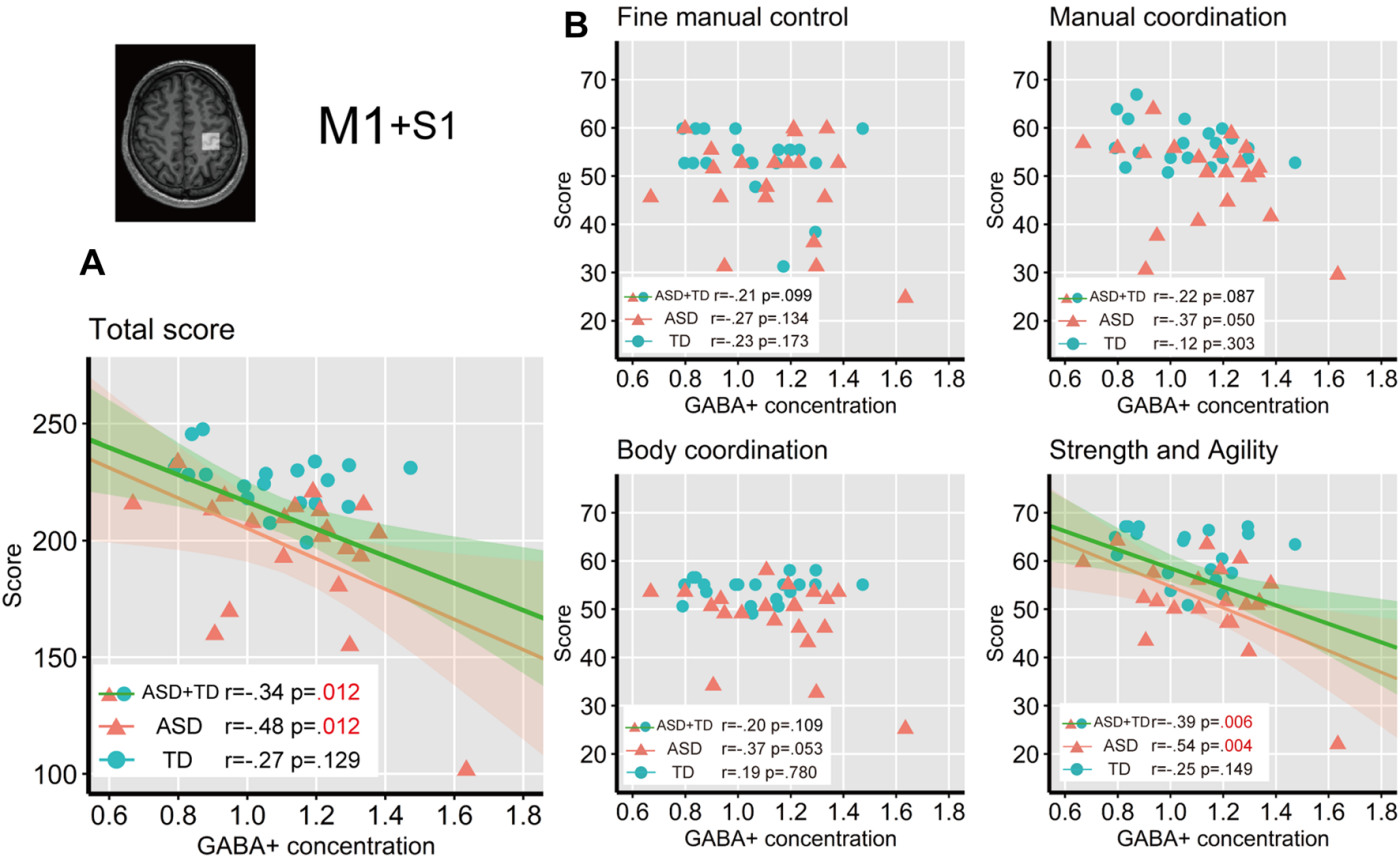
Correlations between tissue-corrected GABA+ concentrations in M1 and BOT-2 scores. The shaded areas (green and pink for ASD + TD and ASD, respectively) denote 95% of the confidence interval for the correlation. **a** Correlation between M1 GABA+ concentration and total BOT-2 score. **b** Correlation between GABA+ concentration in M1 and subcategory BOT-2 scores. Bonferroni correction for multiple comparisons was applied to the correlation analysis (adjusted alpha *p* < 0.0125)

The correlations between GABA+ concentration in M1 and the BOT-2 scores denoted in Fig. 6. There was a significant negative partial correlation between the GABA+ concentration in M1 and the total BOT-2 score for all participants (*r* = − 0.34, *p* = 0.029, 95% CI [− 0.59, − 0.04]), even in the analysis with permutation-based Pearson’s correlation coefficients (*p* = 0.012). We also separately analyzed partial correlations between BOT-2 scores and the GABA+ concentration in M1 for each group. There was a significant correlation between the GABA+ concentrations in M1 and the total BOT-2 score only in the ASD group (*r* = − 0.48, *p* = 0.034, 95% CI [− 0.76, − 0.06]), even when performing permutation test (*p* = 0.012). Bonferroni correction for multiple comparisons was applied to following analysis (adjusted alpha *p* < 0.0125) (see “Statistical Methods” section). We found significant correlations between GABA+ concentration in M1 and the score of the *Strength and agility* subcategory in both of all participants and the ASD group in the permutation test (*r*s = [− 0.39, − 0.54], *p*s = [0.006, 0.004], 95% CIs = {[− 0.62, − 0.09], [− 0.79, − 0.14]}), despite a marginally significant correlation in the parametric correlation analysis (all participants: *p* = 0.014; ASD group: *p* = 0.015). There was no significant correlation between GABA+ concentration in M1 and the scores in the four subcategories in the TD group.

**Fig. 7:**
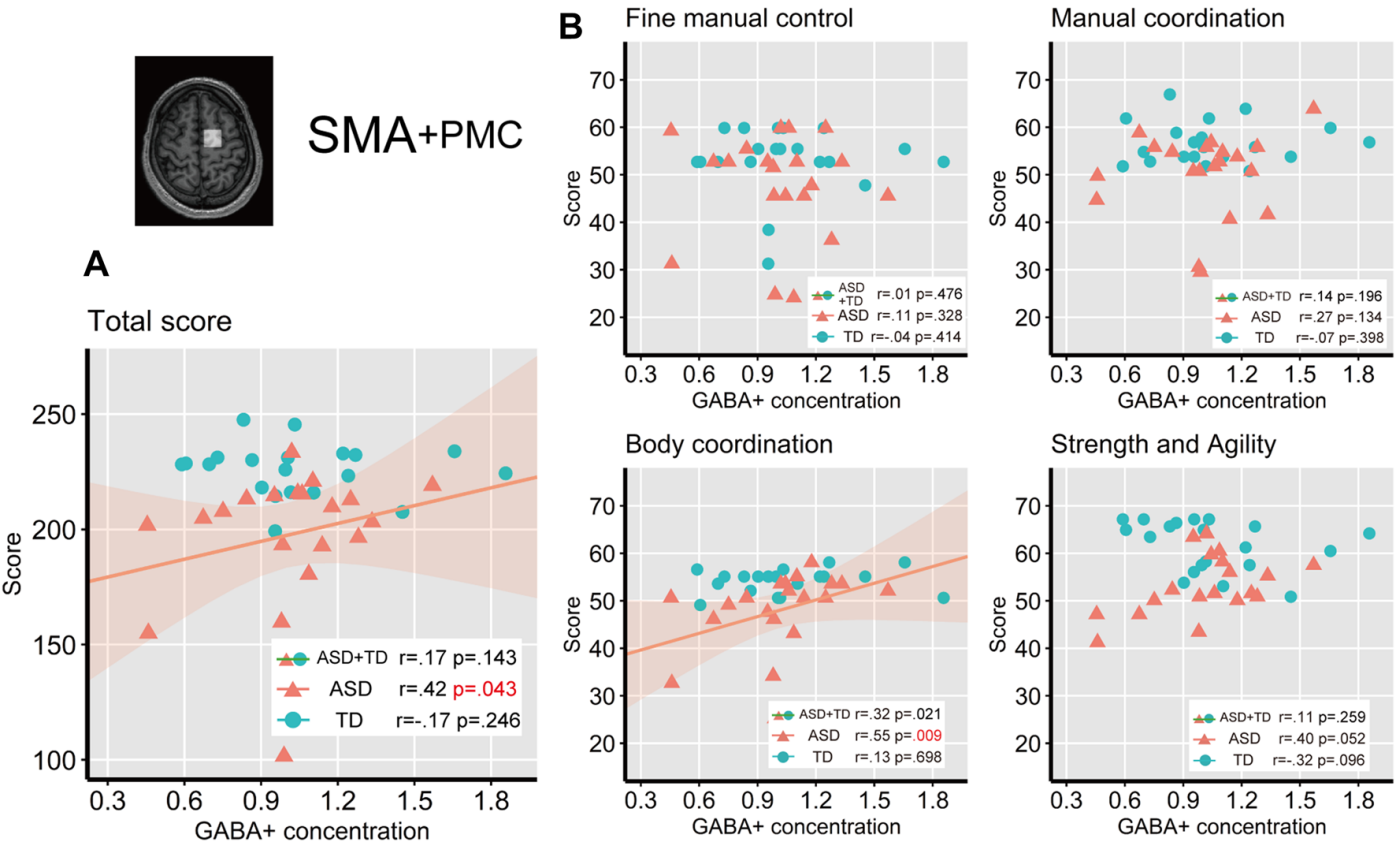
Correlation between tissue-corrected GABA+ concentrations in the SMA and BOT-2 scores. The shaded areas (pink for ASD) denote 95% of the confidence interval for the correlation. **a** Correlation between SMA GABA+ concentration and total BOT-2 score. **b** Correlation between SMA GABA+ concentration and subcategory BOT-2 scores. Bonferroni correction for multiple comparisons was applied to the correlation analysis (adjusted alpha *p* < 0.0125)

The correlations between GABA+ concentration in SMA and the BOT-2 scores denoted in Fig. 7. The GABA+ concentration in SMA was not significantly correlated with the total BOT-2 score for all participants (*r* = 0.17, *p* = 0.289, 95% CI [− 0.15, 0.46]), even when performing permutation test (*p* = 0.143). GABA+ concentration in SMA was significantly positively correlated with the total BOT-2 score in the ASD group in the permutation test (*r* = 0.42, *p* = 0.043, 95% CI [− 0.03, 0.73]), while not significant in the parametric correlation analysis (*p* = 0.073). Bonferroni correction for multiple comparisons was applied to following analysis (adjusted alpha *p* < 0.0125) (see “Statistical Methods” section). We found a significant correlation between the GABA+ concentration in SMA and the score of the *Body coordination* subcategory in the ASD group in the permutation test (*r* = 0.55, *p* = 0.009, 95% CI [0.14, 0.80]), despite a marginally significant correlation in the parametric analysis (*p* = 0.015). There was no significant correlation between GABA+ concentration in SMA and the scores in the four subcategories in the TD group. Supplementary Figs. 3 and 4 show the relationships between the eight subtest scores and GABA+ concentrations in the M1 and SMA.

### Correlation Between AQ Score and GABA+ Concentration

We also investigated the correlations between GABA+ concentrations and AQ scores. There were no significant correlations between them in both M1 and SMA ROIs not only for the pooled data of the ASD and TD groups but also for the data of each group separately (partial correlation with M1 GABA+ in all participants, ASD group and TD group, respectively: *r*s = [0.14, 0.28, − 0.07], *p*s = [0.406, 0.254, 0.766], 95% CIs = {[− 0.18, 0.43], [− 0.17, 0.64], [− 0.50, 0.38]}; partial correlation with SMA GABA+ in all participants, ASD group and TD group: *r*s = [− 0.09, − 0.08, − 0.08], *p*s = [0.578, 0.753, 0.757], 95% CIs = {[− 0.39, 0.23], [− 0.51, 0.38],[ − 0.51, 0.38]}).

## Discussion

Previous studies have reported that decreased GABA+ concentrations (i.e., a weak inhibitor of neural activity) in M1 result in better motor performance in neurotypical participants (Cogiamanian et al. 2007; Stagg et al. 2011; Tanaka et al. 2009). This seemed contradictory to previous findings that decreased GABA+ concentrations in the cerebral cortex, including M1, were reported in individuals with ASD (Gaetz et al. 2014; Puts et al. 2017) and were associated to high comorbidity rates with various types of motor disabilities reported high comorbidity with various types of motor disabilities (Green et al. 2009). Moreover, it is not clear how motor skills required for daily life activities are related to GABA, because previous work has largely measured unusual motor skills that are only relevant to experimental settings (Cogiamanian et al. 2007; Tanaka et al. 2009). Thus, we investigated the types of motor skills evaluated by a clinically valid assessment measure (BOT-2) that are associated with individual levels of GABA+ concentrations in the motor area of the brain in individuals with ASD.

Our main finding is that higher GABA+ concentration in M1 was associated with poorer overall motor performance, especially in the skills of the *Strength and agility*, which was also poorer in individuals with ASD compared to TD according to the BOT-2 assessment. The category *Strength and agility* reflects skills related to the strength of the trunk, as well as the upper and lower body. Previous studies have suggested that the GABA+ concentration in M1 modulates the neural activity and muscle strength. For example, a-tDCS has been found to increase the maximum force and endurance of isometric contraction of specific muscles (Cogiamanian et al. 2007; Tanaka et al. 2009) and simultaneously cause a degradation of GABA+ in the stimulated area (Kim et al. 2014; Stagg et al. 2009), which indicates that decreased GABA in M1 increases muscle strength. These findings suggest that increased GABA in M1 reduces neural activity and results in motor dysfunction that is observed as reduced muscle strength. One previous study reported that lower scores in a clinical motor assessment (Movement assessment battery for children-second edition) associated with impairments of daily living skills (e.g., eats, dresses and household tasks) (Bremer and Cairney 2018), those difficulties of motor skills in daily life may be derived from increased GABA in M1. The fact that GABA+ concentration in M1 was significantly correlated with the score in the *Strength and agility* subcategory in both ASD and TD groups indicates that the underlying neural basis is shared.

Especially our BOT-2 score data raise questions on how the large variability in participants with ASD influenced our results. This variability may be the reason why no significant group differences between ASD and TD were found for GABA+ concentrations in both M1 and SMA. Previous studies have reported that individuals with ASD have lower GABA+ concentrations in M1 (Gaetz et al. 2014; Puts et al. 2017). Gaetz et al. (2014) also showed that older participants with ASD tended to have higher GABA+ /Cr in this region. Furthermore, age-dependent changes in cerebral GABA+ concentrations were reported by many studies (Clement et al. 1987; McQuail et al. 2015; Rowland et al. 2015). Considering that the participants with ASD in studies reporting reduced GABA+ concentrations in M1 were about 10 years old (Gaetz et al. 2014; Puts et al. 2017) whereas those in the present study were around 19 years old, age-dependent variabilities in GABA+ concentrations may be associated with the severity of disabilities in motor skills in adults/adolescents with ASD. Besides, brain organoids derived from induced pluripotent stem cells of patients with ASD facilitate the production of GABAergic inhibitory neurons (Mariani et al. 2015). These results indicate that there are large individual differences in GABA+ concentrations, especially for adults/adolescents with ASD, and this variability may result in motor skills differences.

Our results indicate a positive correlation between GABA+ concentrations in the SMA and the *Body coordination* in the BOT-2 scores of the ASD group. The category *Body coordination* in BOT-2 evaluates skills to maintain body posture, sequential and simultaneous bodily coordination (e.g., standing on one leg, synchronize tapping feet and fingers of opposite sides). The SMA is crucial in coordinated body motion that requires synchronous contralateral hand movements (Cattaneo et al. 2007; Mita et al. 2009). One recent study showed that such contralateral hand movements facilitate synchronized neural oscillations in the SMA across both hemispheres (Hosaka et al. 2015), which may contribute to the communication of timing information that is required for limb movement (Buzsáki and Draguhn 2004). Since GABA_A_ receptor activation considerably contributes to phasic neural activities of interneurons (Traub et al. 2003), the reduction in GABA could result in unsynchronized neural oscillations across the left and right hemispheres.

In general, M1 is thought to be downstream of motor cortices and associate with each body-movement. One might wonder the reason why we observed that the GABA+ concentration in M1 was correlated only with *Strength and agility,* except for the other subcategories (i.e., *Fine manual control*, *Manual coordination,* and *Body coordination*). The assessing items in *Strength and agility* examine simple muscular strength of the whole body, whereas the other three subcategories examine precise and/or rhythmic control of movements to be coordinated using different body parts even in slow movements. Coordinated movements are mainly assessed by the *Body coordination* subcategory in BOT-2, and such movements are known to associate with brain activity in SMA (Cattaneo et al. 2007; Mita et al. 2009). GABA in that brain area has been assumed to play a substantial role in the modulation of oscillatory neural activities as mentioned above (Traub et al. 2003). Considering that GABA+ concentration in SMA linked to score of the *Body coordination* subcategory in ASD participants, these differences between BOT-2 categories that evaluate distinct aspects of motor skills may be the reason that we detected only selective associations with GABA+ concentrations in both M1 and SMA.

We found no correlations between GABA+ concentrations and autistic traits assessed by the AQ score. A previous study reported that the symptomatic autism severity evaluated by the Autism Spectrum Screening Questionnaire was associated with a lower GABA+/Cr ratio in the left anterior cingulate cortex in children with ASD (Brix et al. 2015). The left anterior cingulate cortex is involved in higher cognitive functions such as social (Amodio and Frith 2006) and emotional (Vogt 2005) cognition. By contrast, only a few studies explained the contributions of M1 and/or SMA to social and emotional processing in the human brain. Based on these previous findings, we speculated that the GABA+ concentrations in those brain areas essentially are engaged in body movements.

This is the first study to examine which types of motor skills are affected by alterations of GABA+ concentrations in individuals with ASD. Overall motor skills, especially the skills that require the strength of the upper or lower body, were poorer in ASD participants with increased M1 GABA+ concentrations, and body coordination was indicated to be associated with lower GABA+ concentrations in the SMA. The present findings contribute to the development of objective evaluations of several motor disabilities and accompanied difficulties derived from autistic features.

The findings of this study need to be interpreted in light of some limitations. The MRS voxel used was relatively small (2 × 2 × 2 cm^3^) compared with the voxel used in several previous studies (Brix et al. 2015; Gaetz et al. 2014) (3 × 3 × 3 cm^3^). Although a smaller voxel improves anatomic specificity, it reduces the signal-to-noise ratio. The voxel size used in the current study might result in poorer signal-to-noise ratio compared to that using a larger voxel size. Because we did not control any medication of ASD participants (see Supplementary Table 3 and Fig. 1), it is difficult to mention the medication effects on GABA concentration and motor performances. The medicines taken by eight ASD participants in the present study have been reported to mainly affect dopaminergic and noradrenergic neurotransmitter (Bymaster et al. 2002; Castellanos et al. 1996; Volkow et al. 1994). It still remains a question whether those medicines influence DCD and GABA concentration, while there seemed to be random distribution of individual GABA concentrations. We should examine the medication effects on the present our findings with sufficient numbers of participants for performing statistical analysis in near future. Furthermore, the statistical power of the correlation between GABA+ concentrations and BOT-2 scores was not very large. Considering that GABA modulates neural oscillations (Traub et al. 2003), leading to coordinated body movements (Hosaka et al. 2015), we need to investigate in future studies how the dynamic GABA metabolite working contributes to motor performances. The BOT-2 assessment is usually used to support diagnosis of motor impairments and to plan clinical interventions for the detected problems. Due to these BOT-2 characteristics, low variability in motor skills can be assumed in TD individuals because the BOT-2 tasks are relatively easy for them. Thus, we may not be able to conclude that GABA+ concentrations in brain motor areas are not associated with motor skills in the TD group unless we evaluate more strictly their motor skills.

## Electronic supplementary material

Below is the link to the electronic supplementary material.

Supplementary file1 (DOCX 1017 kb)

## References

[CR1] Adams ILJ, Lust JM, Wilson PH, Steenbergen B (2014). Compromised motor control in children with DCD: A deficit in the internal model? A systematic review. Neuroscience & Biobehavioral Reviews.

[CR2] Amodio DM, Frith CD (2006). Meeting of minds: the medial frontal cortex and social cognition. Nature Reviews Neuroscience.

[CR3] APA (2013). Diagnostic and statistical manual of mental disorders.

[CR4] Barnhart RC, Davenport MJ, Epps SB, Nordquist VM (2003). Developmental coordination disorder. Physical Therapy.

[CR5] Baron-Cohen S, Wheelwright S, Skinner R, Martin J, Clubley E (2001). The Autism-Spectrum Quotient (AQ): Evidence from asperger syndrome/high-functioning autism, males and females, scientists and mathematicians. Journal of Autism and Developmental Disorders.

[CR6] Bhattacharyya PK, Phillips MD, Stone LA, Bermel RA, Lowe MJ (2013). Sensorimotor cortex gamma-aminobutyric acid concentration correlates with impaired performance in patients with MS. American Journal of Neuroradiology.

[CR7] Blakemore S-J, Wolpert DM, Frith CD (1998). Central cancellation of self-produced tickle sensation. Nature Neuroscience.

[CR8] Blicher JU, Near J, Næss-Schmidt E, Stagg CJ, Johansen-Berg H, Nielsen JF, Ho Y-CL (2014). GABA levels are decreased after stroke and GABA changes during rehabilitation correlate with motor improvement. Neurorehabilitation and Neural Repair.

[CR9] Boy F, Evans CJ, Edden RAE, Singh KD, Husain M, Sumner P (2010). Individual differences in subconscious motor control predicted by GABA concentration in SMA. Current Biology.

[CR10] Bremer E, Cairney J (2018). The interrelationship between motor coordination and adaptive behavior in children with autism spectrum disorder. Frontiers in Psychology.

[CR11] Brix, M. K., Ersland, L., Hugdahl, K., Grüner, R., Posserud, M.-B., Hammar, Å., ... Beyer, M. K. (2015). Brain MR spectroscopy in autism spectrum disorder—the GABA excitatory/inhibitory imbalance theory revisited. *Frontiers in Human Neuroscience*. 10.3389/fnhum.2015.00365 10.3389/fnhum.2015.00365 PMC4475903 26157380

[CR12] Buzsáki G, Draguhn A (2004). Neuronal oscillations in cortical networks. Science.

[CR13] Bymaster FP, Katner JS, Nelson DL, Hemrick-Luecke SK, Threlkeld PG, Heiligenstein JH, Perry KW (2002). Atomoxetine increases extracellular levels of norepinephrine and dopamine in prefrontal cortex of rat: A potential mechanism for efficacy in attention deficit/hyperactivity disorder. Neuropsychopharmacology.

[CR14] Castellanos FX, Elia J, Kruesi MJ, Marsh WL, Gulotta CS, Potter WZ, Rapoport JL (1996). Cerebrospinal fluid homovanillic acid predicts behavioral response to stimulants in 45 boys with attention deficit/hyperactivity disorder. Neuropsychopharmacology.

[CR15] Cattaneo L, Fabbri-Destro M, Boria S, Pieraccini C, Monti A, Cossu G, Rizzolatti G (2007). Impairment of actions chains in autism and its possible role in intention understanding. Proceedings of the National Academy of Sciences United States of America.

[CR16] Clement J, Simler S, Ciesielski L, Mandel P, Cabib S, Puglisi-Allegra S (1987). Age-dependent changes of brain GABA levels, turnover rates and shock-induced aggressive behavior in inbred strains of mice. Pharmacology Biochemistry and Behavior.

[CR17] Cogiamanian F, Marceglia S, Ardolino G, Barbieri S, Priori A (2007). Improved isometric force endurance after transcranial direct current stimulation over the human motor cortical areas. European Journal of Neuroscience.

[CR19] Courchesne E, Yeung-Courchesne R, Hesselink JR, Jernigan TL (1988). Hypoplasia of cerebellar vermal lobules VI and VII in autism. New England Journal of Medicine.

[CR20] Crowe TK (1989). Pediatric assessments: A survey of their use by occupational therapists in Northwestern school systems. The Occupational Therapy Journal of Research.

[CR21] Diedrichsen J, Criscimagna-Hemminger SE, Shadmehr R (2007). Dissociating timing and coordination as functions of the cerebellum. The Journal of Neuroscience.

[CR22] Edden RAE, Puts NAJ, Harris AD, Barker PB, Evans CJ (2014). Gannet: A batch-processing tool for the quantitative analysis of gamma-aminobutyric acid–edited MR spectroscopy spectra. Journal of Magnetic Resonance Imaging.

[CR23] Erdfelder E, Faul F, Buchner A (1996). GPOWER: A general power analysis program. Behavior Research Methods, Instruments, & Computers.

[CR24] Fatemi SH, Halt AR, Realmuto G, Earle J, Kist DA, Thuras P, Merz A (2002). Purkinje cell size is reduced in cerebellum of patients with autism. Cellular and Molecular Neurobiology.

[CR25] Floyer-Lea A, Wylezinska M, Kincses T, Matthews PM (2006). Rapid modulation of GABA concentration in human sensorimotor cortex during motor learning. Journal of Neurophysiology.

[CR26] Ford TC, Crewther DP (2016). A comprehensive review of the 1H-MRS metabolite spectrum in autism spectrum disorder. Frontiers in Molecular Neuroscience.

[CR27] Freitag CM, Kleser C, Schneider M, von Gontard A (2007). Quantitative assessment of neuromotor function in adolescents with high functioning autism and asperger syndrome. Journal of Autism and Developmental Disorders.

[CR28] Gaetz W, Bloy L, Wang DJ, Port RG, Blaskey L, Levy SE, Roberts TP (2014). GABA estimation in the brains of children on the autism spectrum: Measurement precision and regional cortical variation. Neuroimage.

[CR29] Gidley Larson JC, Bastian AJ, Donchin O, Shadmehr R, Mostofsky SH (2008). Acquisition of internal models of motor tasks in children with autism. Brain.

[CR30] Green D, Baird G, Barnett AL, Henderson L, Huber J, Henderson SE (2002). The severity and nature of motor impairment in Asperger's syndrome: A comparison with Specific Developmental Disorder of Motor Function. Journal of Child Psychology and Psychiatry.

[CR31] Green D, Charman T, Pickles A, Chandler S, Loucas T, Simonoff E, Baird G (2009). Impairment in movement skills of children with autistic spectrum disorders. Developmental Medicine & Child Neurology.

[CR32] Groppe DM, Urbach TP, Kutas M (2011). Mass univariate analysis of event-related brain potentials/fields I: A critical tutorial review. Psychophysiology.

[CR33] Harada M, Taki MM, Nose A, Kubo H, Mori K, Nishitani H, Matsuda T (2011). Non-invasive evaluation of the GABAergic/glutamatergic system in autistic patients observed by MEGA-editing proton MR spectroscopy using a clinical 3 tesla instrument. Journal of Autism and Developmental Disorders.

[CR34] Harris AD, Puts NA, Edden RA (2015). Tissue correction for GABA-edited MRS: Considerations of voxel composition, tissue segmentation, and tissue relaxations. Journal of Magnetic Resonance Imaging.

[CR35] Hattingen E, Lückerath C, Pellikan S, Vronski D, Roth C, Knake S, Pilatus U (2014). Frontal and thalamic changes of GABA concentration indicate dysfunction of thalamofrontal networks in juvenile myoclonic epilepsy. Epilepsia.

[CR36] Heba S, Puts NAJ, Kalisch T, Glaubitz B, Haag LM, Lenz M, Schmidt-Wilcke T (2015). Local GABA concentration predicts perceptual improvements after repetitive sensory stimulation in humans. Cerebral Cortex.

[CR37] Hosaka, R., Nakajima, T., Aihara, K., Yamaguchi, Y., & Mushiake, H. (2015). Laterality of gamma-oscillations in primate supplementary motor area during performance of visually-guided movements. In *Advances in Cognitive Neurodynamics (IV)* (pp. 165–169). Springer, Dordrecht.

[CR38] Jansen JFA, Backes WH, Nicolay K, Kooi ME (2006). 1H MR spectroscopy of the brain: Absolute quantification of metabolites. Radiology.

[CR39] Jansiewicz EM, Goldberg MC, Newschaffer CJ, Denckla MB, Landa R, Mostofsky SH (2006). Motor signs distinguish children with high functioning autism and asperger’s syndrome from controls. Journal of Autism and Developmental Disorders.

[CR40] Kawato M (1999). Internal models for motor control and trajectory planning. Current Opinion in Neurobiology.

[CR41] Kemper TL, Bauman ML (1993). The contribution of neuropathologic studies to the understanding of autism. Neurologic Clinics.

[CR42] Kim S, Stephenson MC, Morris PG, Jackson SR (2014). tDCS-induced alterations in GABA concentration within primary motor cortex predict motor learning and motor memory: A 7T magnetic resonance spectroscopy study. Neuroimage.

[CR43] Krnjević K, Schwartz S (1967). The action of γ-Aminobutyric acid on cortical neurones. Experimental Brain Research.

[CR45] Losse A, Henderson SE, Elliman D, Hall D, Knight E, Jongmans M (1991). Clumsiness in children: Do they grow out of it? A 10-year follow-up study. Developmental Medicine & Child Neurology.

[CR46] Manjiviona J, Prior M (1995). Comparison of Asperger syndrome and high-functioning autistic children on a Test of Motor Impairment. Journal of Autism and Developmental Disorders.

[CR47] Margari L, De Giacomo A, Craig F, Palumbi R, Peschechera A, Margari M, Dicuonzo F (2018). Frontal lobe metabolic alterations in autism spectrum disorder: A (1)H-magnetic resonance spectroscopy study. Neuropsychiatric disease and treatment.

[CR48] Mariani J, Coppola G, Zhang P, Abyzov A, Provini L, Tomasini L, Vaccarino FM (2015). FOXG1-dependent dysregulation of GABA/glutamate neuron differentiation in autism spectrum disorders. Cell.

[CR49] McQuail JA, Frazier CJ, Bizon JL (2015). Molecular aspects of age-related cognitive decline: The role of GABA signaling. Trends in Molecular Medicine.

[CR50] Mita A, Mushiake H, Shima K, Matsuzaka Y, Tanji J (2009). Interval time coding by neurons in the presupplementary and supplementary motor areas. Nature Neuroscience.

[CR51] Miyahara M, Tsujii M, Hori M, Nakanishi K, Kageyama H, Sugiyama T (1997). Brief report: Motor incoordination in children with asperger syndrome and learning disabilities. Journal of Autism and Developmental Disorders.

[CR52] Mullins PG, McGonigle DJ, O'Gorman RL, Puts NAJ, Vidyasagar R, Evans CJ, Edden RAE (2014). Current practice in the use of MEGA-PRESS spectroscopy for the detection of GABA. Neuroimage.

[CR53] Nakai T, Okanoya K (2016). Individual variability in verbal fluency correlates with gamma-aminobutyric acid concentration in the left inferior frontal gyrus. NeuroReport.

[CR54] Near J, Edden R, Evans CJ, Paquin R, Harris A, Jezzard P (2015). Frequency and phase drift correction of magnetic resonance spectroscopy data by spectral registration in the time domain. Magnetic Resonance in Medicine.

[CR55] Oberman LM, Hubbard EM, McCleery JP, Altschuler EL, Ramachandran VS, Pineda JA (2005). EEG evidence for mirror neuron dysfunction in autism spectrum disorders. Cognitive Brain Research.

[CR56] Oldfield RC (1971). The assessment and analysis of handedness: The Edinburgh inventory. Neuropsychologia.

[CR57] Pizzarelli R, Cherubini E (2011). Alterations of GABAergic signaling in autism spectrum disorders. Neural Plasticity.

[CR58] Puts NAJ, Wodka EL, Harris AD, Crocetti D, Tommerdahl M, Mostofsky SH, Edden RAE (2017). Reduced GABA and altered somatosensory function in children with autism spectrum disorder. Autism Research.

[CR18] R Core Team. (2018). R: A language and environment for statistical computing. Retrieved from https://www.R-project.org/.

[CR59] Reardon S (2016). 'Brain doping' may improve athletes' performance. Nature.

[CR60] Reynolds JE, Licari MK, Billington J, Chen Y, Aziz-Zadeh L, Werner J, Bynevelt M (2015). Mirror neuron activation in children with developmental coordination disorder: A functional MRI study. International Journal of Developmental Neuroscience.

[CR61] Ritvo ER, Freeman BJ, Scheibel AB, Duong T, Robinson H, Guthrie D, Ritvo A (1986). Lower Purkinje cell counts in the cerebella of four autistic subjects: Initial findings of the UCLA-NSAC research report. The American Journal of Psychiatry.

[CR62] Rizzolatti G, Fogassi L, Gallese V (2001). Neurophysiological mechanisms underlying the understanding and imitation of action. Nature Reviews Neuroscience.

[CR63] Robert BH, Brett BD (2005). Bruininks-Oseretsky test of motor proficiency.

[CR64] Rothman DL, Petroff OA, Behar KL, Mattson RH (1993). Localized 1H NMR measurements of gamma-aminobutyric acid in human brain in vivo. Proceedings of the National Academy of Sciences United States of America.

[CR65] Rowland LM, Krause BW, Wijtenburg SA, McMahon RP, Chiappelli J, Nugent KL, Hong LE (2015). Medial frontal GABA is lower in older schizophrenia: A MEGA-PRESS with macromolecule suppression study. Molecular Psychiatry.

[CR66] Sanaei Nezhad F, Anton A, Michou E, Jung J, Parkes LM, Williams SR (2018). Quantification of GABA, glutamate and glutamine in a single measurement at 3 T using GABA-edited MEGA-PRESS. NMR in Biomedicine.

[CR67] Smith MA, Shadmehr R (2005). Intact ability to learn internal models of arm dynamics in huntington's disease but not cerebellar degeneration. Journal of Neurophysiology.

[CR68] Stagg CJ, Best JG, Stephenson MC, O'Shea J, Wylezinska M, Kincses ZT, Johansen-Berg H (2009). Polarity-sensitive modulation of cortical neurotransmitters by transcranial stimulation. The Journal of Neuroscience.

[CR69] Stagg CJ, Bachtiar V, Johansen-Berg H (2011). The role of GABA in human motor learning. Current Biology.

[CR70] Staples KL, Reid G (2010). Fundamental movement skills and autism spectrum disorders. Journal of Autism and Developmental Disorders.

[CR71] Tanaka S, Hanakawa T, Honda M, Watanabe K (2009). Enhancement of pinch force in the lower leg by anodal transcranial direct current stimulation. Experimental Brain Research.

[CR72] Traub RD, Cunningham MO, Gloveli T, LeBeau FEN, Bibbig A, Buhl EH, Whittington MA (2003). GABA-enhanced collective behavior in neuronal axons underlies persistent gamma-frequency oscillations. Proceedings of the National Academy of Sciences Unites States of America.

[CR73] Vega ADL, Brown MS, Snyder HR, Singel D, Munakata Y, Banich MT (2014). Individual differences in the balance of GABA to glutamate in pFC predict the ability to select among competing options. Journal of Cognitive Neuroscience.

[CR74] Vogt BA (2005). Pain and emotion interactions in subregions of the cingulate gyrus. Nature Reviews Neuroscience.

[CR75] Volkow ND, Wang GJ, Fowler JS, Logan J, Schlyer D, Hitzemann R (1994). Imaging endogenous dopamine competition with [11C]raclopride in the human brain. Synapse (New York, N. Y.).

[CR76] Wakabayashi A, Tojo Y, Baron-Cohen S, Wheelwright S (2004). The Autism-Spectrum Quotient(AQ)Japanese version: Evidence from high-functioning clinical group and normaladults. The Japanese Journal of Psychology.

[CR77] Yousry TA, Schmid UD, Alkadhi H, Schmidt D, Peraud A, Buettner A, Winkler P (1997). Localization of the motor hand area to a knob on the precentral gyrus A new landmark. Brain.

