# Supplementary material for: Altered GABA Concentration in Brain Motor Area Is Associated with the Severity of Motor Disabilities in Individuals with Autism Spectrum Disorder
Source: J Autism Dev Disord. 2020 Jan 30;50(8):2710–22. doi: 10.1007/s10803-020-04382-x (PMC7374467; doi:10.1007/s10803-020-04382-x)
Supplement: Supplementary file 1 — Supplementary file1 (DOCX 1017 kb) [file 10803_2020_4382_MOESM1_ESM.docx]

**Journal of Autism Developmental Disorders:**

**Altered GABA concentration in brain motor area is associated with the severity of motor disabilities in individuals with autism spectrum disorder**

Table of Contents

1. Supplementary Table 1
2. Supplementary Table 2
3. Supplementary Table 3
4. Supplementary Table 4
5. Supplementary Table 5
6. Supplementary Fig. 1
7. Supplementary Fig. 2
8. Supplementary Fig. 3
9. Supplementary Fig. 4

Supplementary Table 1 Information of the intelligence quotient assessed using the Wechsler Intelligence Scale for Children-Fourth Edition (WISC-IV).

|  | Sex | Age* | VCI | PRI | WMI | PSI | FIQ |
| --- | --- | --- | --- | --- | --- | --- | --- |
| Subj1 | F | 15 | 80 | 91 | 72 | 88 | 86 |
| Subj2 | F | 17 | 121 | 58 | 88 | 61 | 78 |

*: Age at the experiment day

Abbreviations: M, male; F, female; VCI, verbal comprehension index; PRI, perceptual reasoning index; WMI, working memory index; PSI, processing speed index; FIQ, full-scale intelligence quotient.

|  | Sex | Age* | VIQ | PIQ | FIQ |
| --- | --- | --- | --- | --- | --- |
| Subj3 | M | 15 | 79 | 103 | 89 |

Supplementary Table 2 Information of the intelligence quotient assessed using the Wechsler Intelligence Scale for Children-Third Edition (WISC-Ⅲ).

Abbreviations: VIQ, verbal intelligence quotient; PIQ, performance intelligence quotient; FIQ, full-scale intelligence quotient.

The WISC-IV is an assessment tool of Intelligence Quotients (IQs) of persons under 15 years old. It was used to evaluate one 15-year-old participants and one 17-year-old participant who were assessed IQs seven years and three years ago, respectively. In addition, the WISC-Ⅲ (previous version of WISC- IV) was used for evaluation of one 15-year-old participant. All participants had FIQ above 75.

| ID | Sex | Age | LQ | AQ | GABA+ conc (M1) | GABA+ conc (SMA) | Medications |
| --- | --- | --- | --- | --- | --- | --- | --- |
| ASD1 | M | 19 | 100 | 28 | 1.211 | 1.250 | None |
| ASD2 | M | 20 | 63 | 33 | 1.138 | 0.951 | Atomoxetine hydrochloride |
| ASD3 | M | 21 | -53 | 21 | 1.105 | 1.138 | None |
| ASD4 | M | 15 | 67 | 34 | 1.329 | 0.984 | None |
| ASD5 | M | 19 | 89 | 13 | 0.669 | 1.043 | None |
| ASD6 | M | 21 | 80 | 34 | 1.265 | 1.086 | None |
| ASD7 | M | 20 | 60 | 22 | 1.297 | 0.459 | None |
| ASD8 | M | 20 | 75 | 27 | 1.337 | 1.060 | N/A |
| ASD9 | F | 23 | 100 | 29 | 1.108 | 1.178 | None |
| ASD10 | M | 15 | 89 | 33 | 0.934 | 1.570 | Methylphenidate hydrochloride,  Modafinil |
| ASD11 | M | 19 | 56 | 29 | 1.380 | 1.333 | None |
| ASD12 | F | 23 | 100 | 38 | 0.906 | 0.980 | N/A |
| ASD13 | M | 17 | 100 | 33 | 1.231 | 0.673 | None |
| ASD14 | M | 25 | 100 | 42 | 1.014 | 0.751 | Methylphenidate hydrochloride,  Guanfacine Hydrochloride |
| ASD15 | M | 19 | 90 | 38 | 0.898 | 0.843 | Methylphenidate hydrochloride,  Atomoxetine hydrochloride,  Duloxetine Hydrochloride |
| ASD16 | F | 18 | 100 | 26 | 0.948 | - | Methylphenidate hydrochloride,  Sulpiride |
| ASD17 | M | 21 | 80 | 31 | 0.798 | 1.019 | Carbamazepine, Levetiracetam |
| ASD18 | F | 17 | 100 | 42 | 1.635 | 0.989 | Methylphenidate Hydrochloride |
| ASD19 | M | 15 | 100 | 24 | 1.190 | 1.102 | None |
| ASD20 | F | 15 | 90 | 37 | 1.287 | 1.280 | Epinephrine |
| ASD21 | F | 22 | 100 | 37 | 1.216 | 0.454 | None |

Supplementary Table 3 Medications taken by ASD participants.

Supplementary Table 4 Average GABA+ concentrations relative to Cr and H_2_O in M1 and SMA in ASD and TD groups.

|  | | ASD group | TD group |
| --- | --- | --- | --- |
| M1 ROI | GABA+/Cr (ns) | 0.097 (±0.018) | 0.089 (±0.014) |
|  | GABA+/H_2_O (ns) | 1.038 (±0.185) | 0.957 (±0.173) |
| SMA ROI | GABA+/Cr (ns) | 0.089 (±0.018) | 0.096 (±0.023) |
|  | GABA+/H_2_O (ns) | 0.942 (±0.258) | 1.006 (±0.323) |

Supplementary Table 5 Correlation coefficients of tissue-corrected GABA+ concentration, GABA+/Cr and GABA+/H_2_O in M1 and SMA.

|  | Tissue-corrected GABA+ | GABA+/Cr | GABA+/H_2_O |
| --- | --- | --- | --- |
| Tissue-corrected GABA+ | ― | 0.86** (M1)  0.88** (SMA) | 0.96** (M1)  0.99** (SMA) |
| GABA+/Cr | ― | ― | 0.90** (M1)  0.90** (SMA) |
| GABA+/H_2_O | ― | ― | ― |

** *p* < 0.01


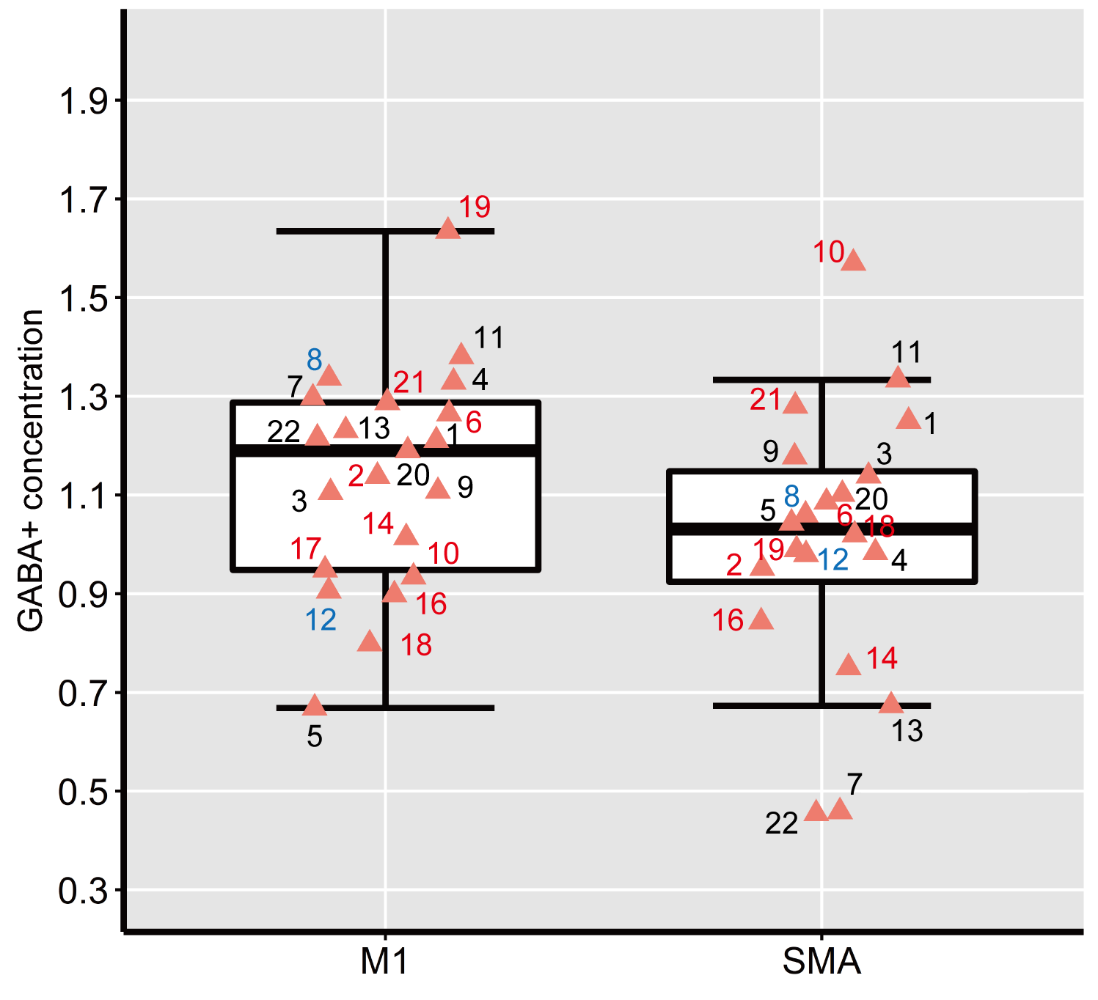


Supplementary Fig. 1 Tissue-corrected GABA+ concentrations in M1 and SMA in the ASD group. Labels indicates each subject’s ID corresponding to that in the Supplementary Table 3. The labels colored in red indicates subjects who have taken medications prior to the day we measured MRS, while labels colored in blue indicates subjects who were difficult to obtain information of taking medicines.


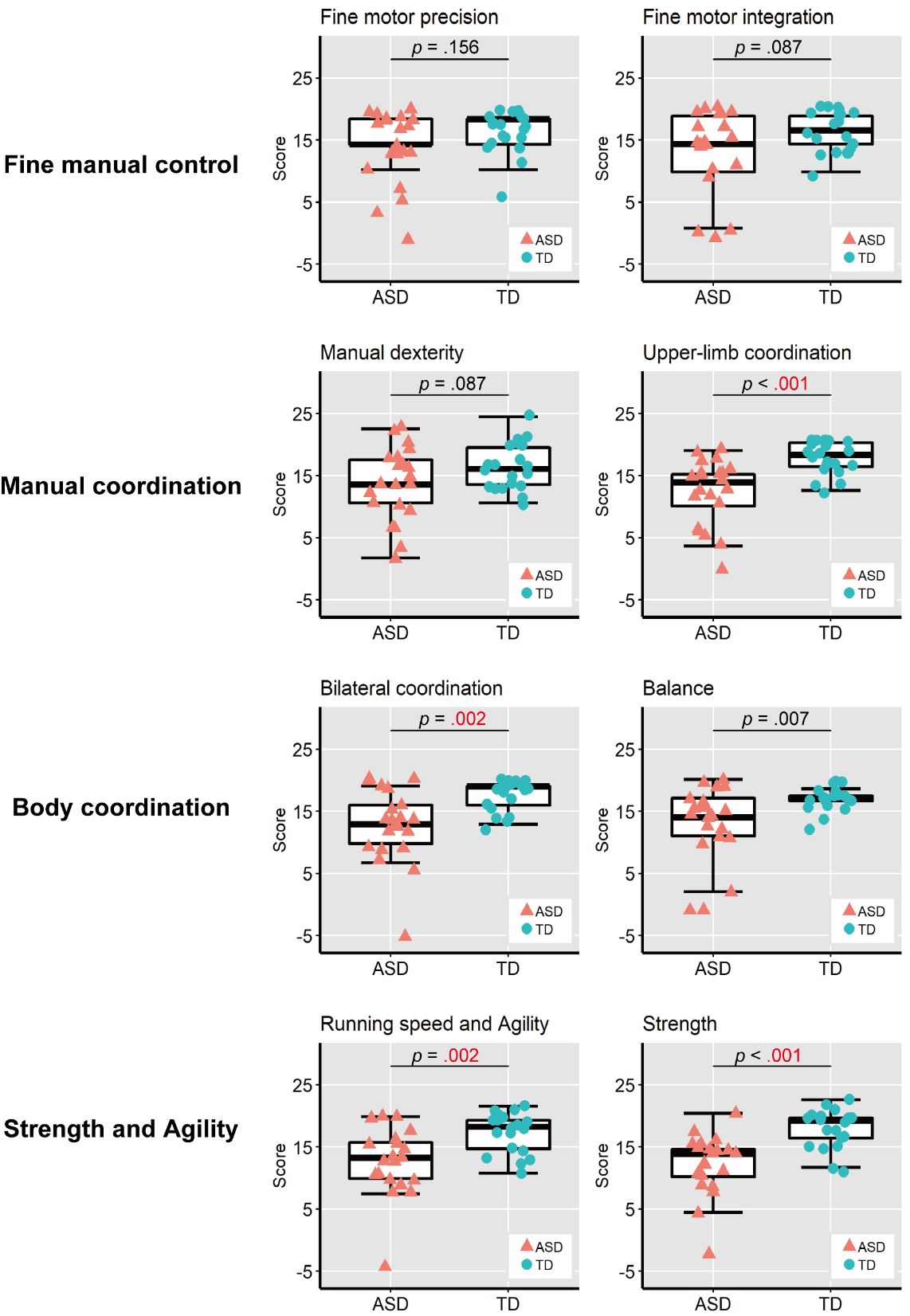


Supplementary Fig. 2 Eight subtests’ scores in the BOT-2 both in the ASD and TD groups. Labels of four sub-categories which each subtest is assigned are shown as bold texts. Bonferroni correction for multiple comparisons was applied to the correlation analysis (adjusted alpha *p* < 0.00625). There were significant between-group differences as regard to the subtests of *upper-limb coordination*, *bilateral coordination*, *running speed and agility* and *strength*.


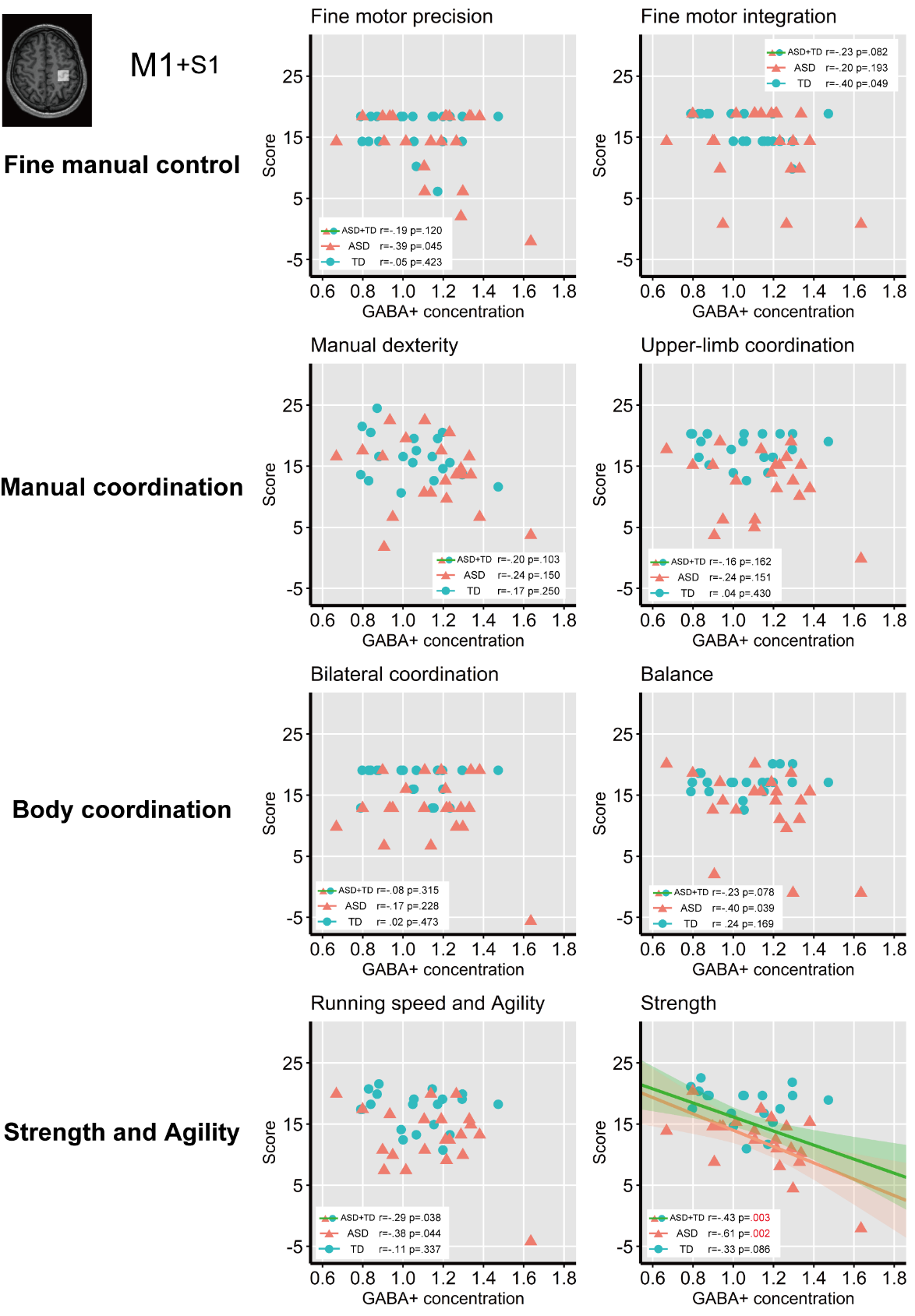


Supplementary Fig. 3 Correlation between GABA+ concentrations in M1 and subtests’ score in the BOT-2. Bonferroni correction for multiple comparisons was applied to the correlation analysis (adjusted alpha *p* < 0.00625). There were negative significant correlations as regard to subtests of *Strength* in ASD + TD group and only in ASD group.


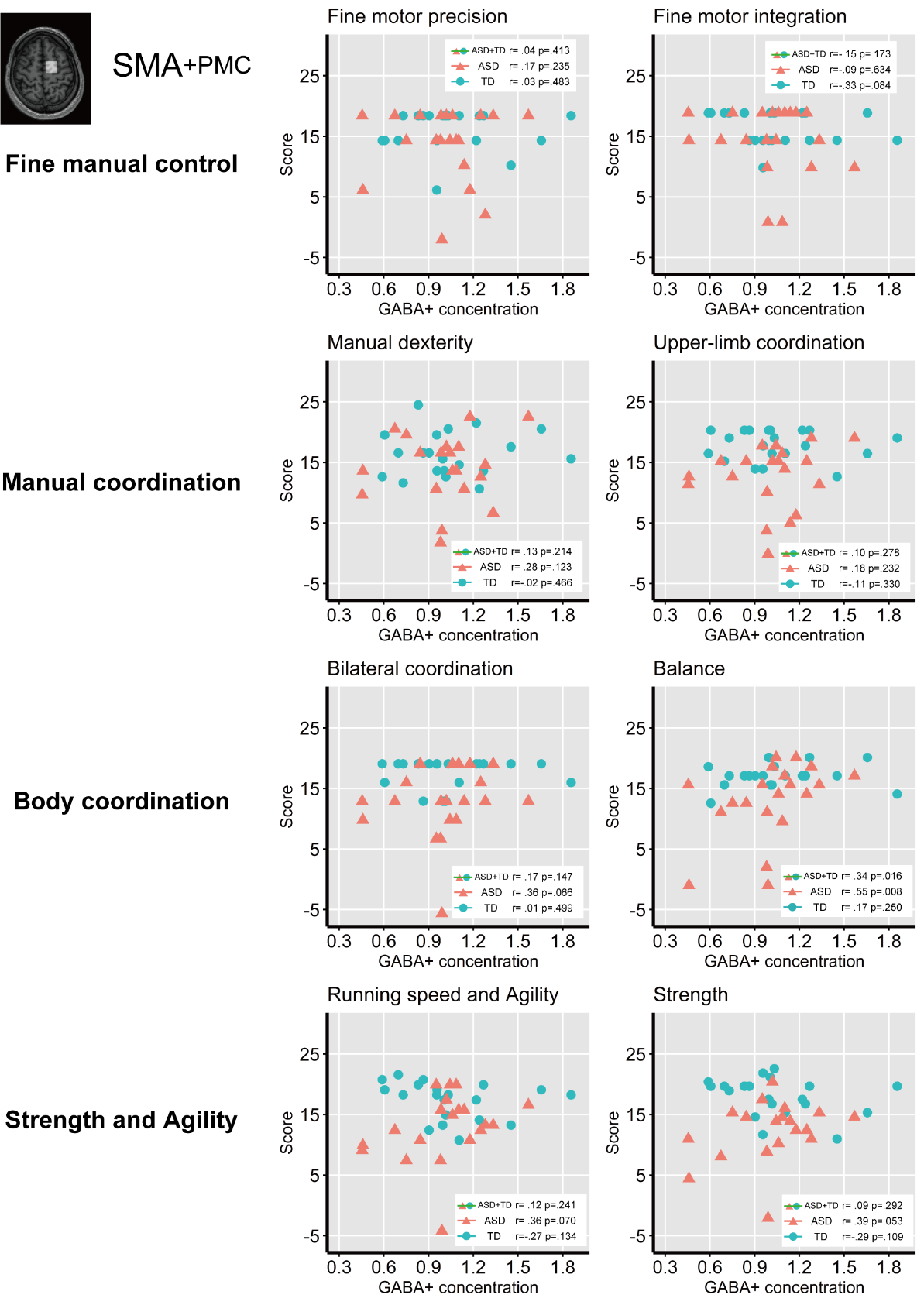


Supplementary Fig. 4 Correlation between GABA+ concentrations in SMA and subtests’ score in the BOT-2. Bonferroni correction for multiple comparisons was applied to the correlation analysis (adjusted alpha *p* < 0.00625). There were no significant correlations while the score of *Balance* were marginally significantly correlated with the GABA+ concentration in ASD group.
